# Poly[hexa­aqua­hexa­kis­(μ-pyridine-2,4-dicarboxyl­ato)tricopper(II)dieuropium(III)]

**DOI:** 10.1107/S1600536810044594

**Published:** 2010-11-06

**Authors:** Shie Fu Lush, Fwu Ming Shen

**Affiliations:** a Department of General Education Center, Yuanpei University, HsinChu, 30015 Taiwan; b Department of Biotechnology, Yuanpei University, No. 306, Yuanpei St, HsinChu, 30015 Taiwan

## Abstract

The asymmetric unit of the title heterometallic coordination polymer, [Cu_3_Eu_2_(C_7_H_3_NO_4_)_6_(H_2_O)_6_]_*n*_, contains one Eu^III^ and two Cu^II^ atoms, three pyridine-2,4-dicarboxylate (pdc)^2−^ anions and three water molecules. One Cu^II^ atom is located on an inversion center and is *N*,*O*-chelated by two pdc^2−^ anions in the equatorial plane and further coordinated by two carboxyl­ate O atoms from another two pdc anions in the axial positions, with an elongated octa­hedral geometry [Cu—O = 2.409 (3) Å in the axial direction]; the other Cu atom is *N*,*O*-chelated by two pdc anions in the coordination basal plane and coordinated by a carboxyl O atom at the apical position with a distorted square-pyramidal geometry [Cu—O = 2.359 (3) Å in the apical direction]. The Eu atom is eight-coordinated with a distorted square-anti­prismatic geometry formed by five carboxyl­ate O atoms from five pdc anions and three water mol­ecules. The carboxyl­ate anions bridge adjacent Eu and Cu atoms, forming the coordination polymer. Inter- and intra­molecular O—H⋯O hydrogen bonding occurs in the structure. π–π stacking further consolidates the crystal structure, the centroid–centroid distance between parallel pyridine rings being 3.367 (2) Å.

## Related literature

For structures and applications of related heterometallic lanthanide-transition metal coordination polymers, see: Huang *et al.* (2008*a* ▶,*b* ▶). For the coordination modes of the pyridine-2,6-dicarboxyl­ate ligand, see: Ma *et al.* (2010 ▶); Zhao *et al.* (2007 ▶); Wang *et al.* (2007 ▶). For the coordination modes of the pyridine-2,5-dicarboxyl­ate ligand, see: Song *et al.* (2006 ▶); Wang *et al.* (2009 ▶). For the coordination modes of the pyridine-2,3-dicarboxyl­ate ligand, see: Wang *et al.* (2010 ▶).
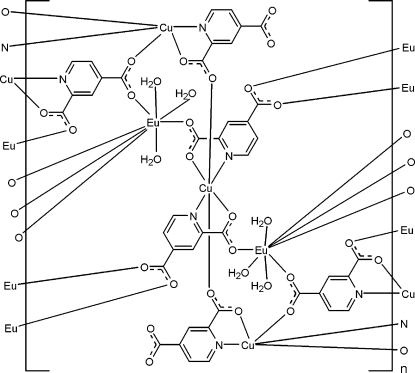


## Experimental

### 

#### Crystal data

[Cu_3_Eu_2_(C_7_H_3_NO_4_)_6_(H_2_O)_6_]
*M* *_r_* = 1593.29
Triclinic, 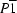

*a* = 9.4296 (10) Å
*b* = 10.7002 (11) Å
*c* = 12.2874 (13) Å
α = 86.186 (2)°
β = 81.556 (2)°
γ = 86.561 (2)°
*V* = 1222.0 (2) Å^3^
*Z* = 1
Mo *K*α radiation
μ = 3.92 mm^−1^
*T* = 294 K
0.24 × 0.20 × 0.20 mm

#### Data collection

Bruker SMART CCD area-detector diffractometer
Absorption correction: multi-scan (*SADABS*; Bruker, 2000 ▶) *T*_min_ = 0.659, *T*_max_ = 0.977
12982 measured reflections
5780 independent reflections
4828 reflections with *I* > 2σ(*I*)
*R*_int_ = 0.044

#### Refinement

*R*[*F* ^2^ > 2σ(*F* ^2^)] = 0.028
*wR*(*F* ^2^) = 0.066
*S* = 0.98
5780 reflections
376 parameters
H-atom parameters constrained
Δρ_max_ = 1.69 e Å^−3^
Δρ_min_ = −0.94 e Å^−3^

### 

Data collection: *SMART* (Bruker, 2000 ▶); cell refinement: *SAINT* (Bruker, 1999 ▶); data reduction: *SAINT*; program(s) used to solve structure: *SHELXTL* (Sheldrick, 2008 ▶); program(s) used to refine structure: *SHELXTL*; molecular graphics: *PLATON* (Spek, 2009 ▶); software used to prepare material for publication: *PLATON*.

## Supplementary Material

Crystal structure: contains datablocks global, I. DOI: 10.1107/S1600536810044594/xu5066sup1.cif

Structure factors: contains datablocks I. DOI: 10.1107/S1600536810044594/xu5066Isup2.hkl

Additional supplementary materials:  crystallographic information; 3D view; checkCIF report

## Figures and Tables

**Table 1:** Selected bond lengths (Å)

Eu1—O6^i^	2.485 (2)
Eu1—O8	2.386 (3)
Eu1—O10^ii^	2.404 (2)
Eu1—O11^iii^	2.385 (2)
Eu1—O12^i^	2.328 (2)
Eu1—O13	2.454 (3)
Eu1—O14	2.442 (3)
Eu1—O15	2.510 (3)
Cu1—O1	1.932 (3)
Cu1—O5^iv^	1.944 (2)
Cu1—O7	2.359 (3)
Cu1—N1	1.975 (3)
Cu1—N2^iv^	1.975 (3)
Cu2—O2	2.409 (3)
Cu2—O9	1.968 (2)
Cu2—N3	1.969 (3)

Symmetry codes: (i) 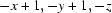; (ii) 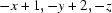; (iii) 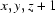; (iv) 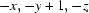.

**Table 2:** Hydrogen-bond geometry (Å, °)

*D*—H⋯*A*	*D*—H	H⋯*A*	*D*⋯*A*	*D*—H⋯*A*
O13—H13*A*⋯O5^i^	0.82	2.00	2.769 (3)	155
O13—H13*B*⋯O15^v^	0.82	2.02	2.822 (4)	164
O14—H14*A*⋯O7	0.82	1.89	2.690 (4)	164
O14—H14*B*⋯O4^vi^	0.82	1.88	2.667 (4)	161
O15—H15*A*⋯O3^vii^	0.82	1.83	2.630 (4)	164
O15—H15*B*⋯O4^vi^	0.82	2.06	2.814 (4)	153

Symmetry codes: (i) 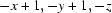; (v) 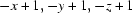; (vi) 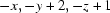; (vii) 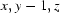.

**Fig. 1.**
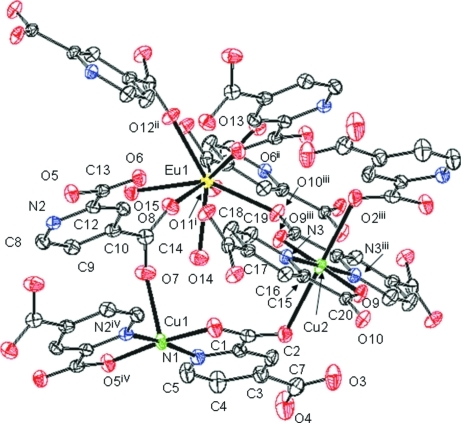
View of the title compound with the atom numbering scheme. Displacement ellipsoids for non-H atoms are drawn at the 50% probability level. H atoms have been omitted for clarity. [Symmetry codes: (i) x, y, z + 1; (ii) -x + 1, -y + 1, -z; (iii) -x + 1, -y + 2, -z; (iv) -x, -y + 1, -z].

**Fig. 2.**
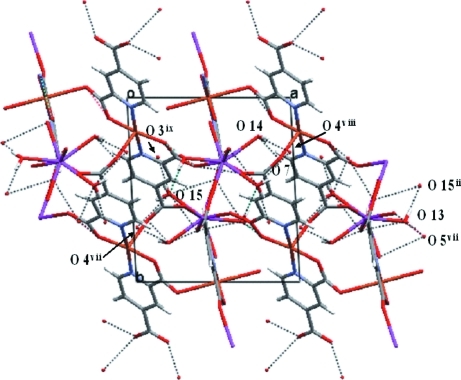
The molecular packing for the title compound, viewed along the c axis. Hydrogen bonds are shown as dashed lines.

## supplementary crystallographic information

### Comment

In recent years, many research groups have devoted their work to the design and synthesis of lanthanide–transition(3 d-4f) heterometallic coordination frameworks with bridging multifunctional organic ligands, such as pyridinedicarboxylic acid (Ma *et al.*, 2010; Song *et al.*, 2006; Wang *et al.*,2007; Wang *et al.*, 2009; Wang *et al.*, 2010; Zhao *et al.*,2007). Pyridine-2,4-dicarboxylic acid (pdaH_2_) ligand is a good candidate due to its flexible and various coordination donors containing either N– or O–atom donors. Some examples of coordination with pdaH_2_ have been reported (Huang *et al.*, 2008*a*; Huang *et al.*, 2008*b*.).

Herein we report a new 3 d-4f heterometallic coordination polymer based on pdaH_2_ ligand, formulated as [Eu_2_Cu_3_(C_7_H_3_NO_4_)_6_(H_2_O)_6_]_n_. The symmetric unit of the title compound contains one octa-coordinated Eu^III^ atom and two types of environments of Cu^II^ center, the penta-coordinate Cu^II^ atom is in a square- pyramidal geometry, the other Cu^II^ atom is in a slightly distorted octahedral geometry. The Eu^III^ ion presents a EuO_8_ square antiprismatic coordination geometry, formed by five mono-dentate pda O atoms and three coordinate water molecules, the Cu1^II^ion presents a CuN_2_O_3_ square- pyramidal coordination geometry, formed by two bidentate pda (–NO–) ligand (2-carboxy) in the equatorial plane, and one O atom from monodentate pda (4-carboxy) ligand at the axial sites, the other Cu2^II^ ion presents a slightly distorted (CuN_2_O_4_) octahedral coordination geometry, formed by two bidentate pda (–NO–) ligand (2-carboxy) in the equatorial plane, and two monodentate pda(2-carboxy) ligands at the axial sites (Table 1, Fig 1). The pda One carboxyl group bridges the neighboring Eu or Cu cations, forming the 3-D polymeric architecture.

In the title crystal structure, a three-dimensional network is formed *via* intra- intermolecular O—H···O hydrogen bonds (Table 2, Fig. 2). In addition, C—H···O hydrogen bonds (full details and symmetry codes are given in Table 2), C7—O3···*Cg*8 (N3/C15—C19) interactions are also present. The π···π, π···Metal stacking interactions are also observed, the centroid-centroid distance between the pyridine rings being 3.367 (2)Å [C*g*7^iv^···C*g*7 (N2/C8—C12)] [symmetry codes: -*x*, 1 - *y*, -*z*]. The C*g*1 (Cu1/O1—N1—C1—C6)···Cu1 interaction is 3.905Å [symmetry codes: -*x*, 2 - *y*, -*z*].

### Experimental

A mixture of europium chloride hexahydrate (0.25 mmol, 0.0916 g), copper acetate hydrate (0.25 mmol, 0.050 g), pyridine-2,4-dicarboxylic acid (0.25 mmol, 0.0418 g) and 10 ml H_2_O were put in a 23-ml Teflon liner reactor and heated at 418 K in oven for 48 h. The resulting solution was slowly cooled to room temperature. The blue transparent single crystals of the title complex were obtained in 43.21% yield (based on Eu).

### Refinement

Water H atoms wre located in a difference Fourier map and refined with the distances constraints of O—H = 0.82 Å, *U*_iso_(H) = 1.5*U*_eq_(O). Other H atoms were positioned geometrically with C—H = 0.93 Å (aromatic), and refined using a riding model with *U*_iso_(H) = 1.2*U*_eq_(C).

### Crystal data

**Table:**

[Cu_3_Eu_2_(C_7_H_3_NO_4_)_6_(H_2_O)_6_]	*Z* = 1
*M**_r_* = 1593.29	*F*(000) = 777
Triclinic, *P*1	*D*_x_ = 2.165 Mg m^−^^3^
Hall symbol: -P 1	Mo *K*α radiation, λ = 0.71073 Å
*a* = 9.4296 (10) Å	Cell parameters from 6583 reflections
*b* = 10.7002 (11) Å	θ = 2.5–25.0°
*c* = 12.2874 (13) Å	µ = 3.92 mm^−^^1^
α = 86.186 (2)°	*T* = 294 K
β = 81.556 (2)°	Equant, blue
γ = 86.561 (2)°	0.24 × 0.20 × 0.20 mm
*V* = 1222.0 (2) Å^3^	

### Data collection

**Table:**

Bruker SMART CCD area-detector diffractometer	5780 independent reflections
Radiation source: fine-focus sealed tube	4828 reflections with *I* > 2σ(*I*)
graphite	*R*_int_ = 0.044
Detector resolution: 9 pixels mm^-1^	θ_max_ = 28.3°, θ_min_ = 1.7°
φ and ω scans	*h* = −12→12
Absorption correction: multi-scan (*SADABS*; Bruker, 2000)	*k* = −13→13
*T*_min_ = 0.659, *T*_max_ = 0.977	*l* = −15→16
12982 measured reflections	

### Refinement

**Table:**

Refinement on *F*^2^	Primary atom site location: structure-invariant direct methods
Least-squares matrix: full	Secondary atom site location: difference Fourier map
*R*[*F*^2^ > 2σ(*F*^2^)] = 0.028	Hydrogen site location: inferred from neighbouring sites
*wR*(*F*^2^) = 0.066	H-atom parameters constrained
*S* = 0.98	*w* = 1/[σ^2^(*F*_o_^2^) + (0.0323*P*)^2^] where *P* = (*F*_o_^2^ + 2*F*_c_^2^)/3
5780 reflections	(Δ/σ)_max_ = 0.001
376 parameters	Δρ_max_ = 1.69 e Å^−^^3^
0 restraints	Δρ_min_ = −0.94 e Å^−^^3^

### Special details

**Table:**

Geometry. Bond distances, angles *etc*. have been calculated using the rounded fractional coordinates. All su's are estimated from the variances of the (full) variance-covariance matrix. The cell e.s.d.'s are taken into account in the estimation of distances, angles and torsion angles
Refinement. Refinement on *F*^2^ for ALL reflections except those flagged by the user for potential systematic errors. Weighted *R*-factors *wR* and all goodnesses of fit *S* are based on *F*^2^, conventional *R*-factors *R* are based on *F*, with *F* set to zero for negative *F*^2^. The observed criterion of *F*^2^ > σ(*F*^2^) is used only for calculating -*R*-factor-obs *etc*. and is not relevant to the choice of reflections for refinement. *R*-factors based on *F*^2^ are statistically about twice as large as those based on *F*, and *R*-factors based on ALL data will be even larger.

### Fractional atomic coordinates and isotropic or equivalent isotropic displacement parameters (Å^2^)

**Table:**

	*x*	*y*	*z*	*U*_iso_*/*U*_eq_	
Eu1	0.42924 (2)	0.65169 (1)	0.35169 (1)	0.0161 (1)	
Cu1	−0.04296 (4)	0.81387 (4)	0.11987 (3)	0.0220 (1)	
Cu2	0.50000	1.00000	0.00000	0.0188 (2)	
O1	0.1188 (3)	0.8879 (2)	0.0288 (2)	0.0274 (8)	
O2	0.2474 (3)	1.0583 (2)	0.0150 (2)	0.0272 (8)	
O3	0.1716 (3)	1.3256 (3)	0.3467 (2)	0.0406 (10)	
O4	−0.0222 (3)	1.2972 (3)	0.4696 (3)	0.0524 (11)	
O5	0.2216 (2)	0.2480 (2)	−0.19845 (19)	0.0238 (7)	
O6	0.3969 (2)	0.3742 (2)	−0.18098 (19)	0.0236 (8)	
O7	0.0882 (3)	0.6635 (2)	0.2196 (2)	0.0313 (8)	
O8	0.3149 (3)	0.5865 (2)	0.2057 (2)	0.0258 (8)	
O9	0.5336 (3)	1.1177 (2)	−0.13010 (19)	0.0230 (7)	
O10	0.5037 (3)	1.1358 (2)	−0.30699 (19)	0.0235 (7)	
O11	0.3895 (3)	0.7209 (2)	−0.46529 (19)	0.0254 (8)	
O12	0.4790 (3)	0.5533 (2)	−0.3775 (2)	0.0269 (8)	
O13	0.6647 (3)	0.6702 (3)	0.4101 (2)	0.0318 (9)	
O14	0.2105 (3)	0.7873 (2)	0.3621 (2)	0.0285 (8)	
O15	0.2311 (3)	0.5207 (2)	0.4481 (2)	0.0262 (8)	
N1	−0.0265 (3)	0.9489 (3)	0.2186 (2)	0.0222 (9)	
N2	0.0738 (3)	0.3055 (3)	−0.0108 (2)	0.0190 (8)	
N3	0.4749 (3)	0.8802 (3)	−0.1097 (2)	0.0179 (8)	
C1	0.0834 (3)	1.0218 (3)	0.1776 (3)	0.0193 (10)	
C2	0.1182 (4)	1.1211 (3)	0.2322 (3)	0.0232 (10)	
C3	0.0356 (4)	1.1510 (3)	0.3314 (3)	0.0260 (11)	
C4	−0.0795 (4)	1.0771 (4)	0.3712 (3)	0.0344 (12)	
C5	−0.1064 (4)	0.9764 (4)	0.3138 (3)	0.0314 (12)	
C6	0.1590 (4)	0.9878 (3)	0.0650 (3)	0.0213 (10)	
C7	0.0646 (4)	1.2678 (4)	0.3881 (3)	0.0282 (12)	
C8	−0.0144 (4)	0.3380 (3)	0.0799 (3)	0.0214 (10)	
C9	0.0190 (4)	0.4295 (3)	0.1443 (3)	0.0225 (10)	
C10	0.1511 (4)	0.4835 (3)	0.1200 (3)	0.0200 (10)	
C11	0.2450 (3)	0.4447 (3)	0.0287 (3)	0.0199 (10)	
C12	0.2009 (3)	0.3591 (3)	−0.0364 (3)	0.0177 (9)	
C13	0.2824 (3)	0.3236 (3)	−0.1467 (3)	0.0187 (10)	
C14	0.1878 (4)	0.5861 (3)	0.1875 (3)	0.0209 (10)	
C15	0.4789 (3)	0.9361 (3)	−0.2116 (3)	0.0177 (9)	
C16	0.4629 (4)	0.8712 (3)	−0.3016 (3)	0.0195 (10)	
C17	0.4471 (4)	0.7427 (3)	−0.2868 (3)	0.0193 (10)	
C18	0.4458 (4)	0.6855 (3)	−0.1819 (3)	0.0248 (10)	
C19	0.4564 (4)	0.7580 (3)	−0.0948 (3)	0.0233 (10)	
C20	0.5063 (3)	1.0751 (3)	−0.2176 (3)	0.0178 (9)	
C21	0.4380 (4)	0.6657 (3)	−0.3837 (3)	0.0195 (10)	
H2A	0.19630	1.16800	0.20320	0.0280*	
H4A	−0.13820	1.09550	0.43650	0.0410*	
H5A	−0.18220	0.92660	0.34220	0.0380*	
H8A	−0.10000	0.29790	0.09970	0.0260*	
H9A	−0.04660	0.45500	0.20380	0.0270*	
H11A	0.33600	0.47600	0.01220	0.0240*	
H13A	0.71830	0.70010	0.35740	0.0480*	
H13B	0.70360	0.60960	0.44030	0.0480*	
H14A	0.16690	0.76300	0.31500	0.0430*	
H14B	0.15520	0.77690	0.41960	0.0430*	
H15A	0.19680	0.46590	0.41800	0.0390*	
H15B	0.15630	0.55220	0.48000	0.0390*	
H16A	0.46260	0.91230	−0.37070	0.0230*	
H18A	0.43800	0.59920	−0.17040	0.0300*	
H19A	0.45030	0.72020	−0.02400	0.0280*	

### Atomic displacement parameters (Å^2^)

**Table:**

	*U*^11^	*U*^22^	*U*^33^	*U*^12^	*U*^13^	*U*^23^
Eu1	0.0202 (1)	0.0122 (1)	0.0166 (1)	−0.0010 (1)	−0.0040 (1)	−0.0033 (1)
Cu1	0.0214 (2)	0.0212 (2)	0.0238 (2)	−0.0061 (2)	0.0003 (2)	−0.0088 (2)
Cu2	0.0269 (3)	0.0140 (3)	0.0167 (3)	−0.0032 (2)	−0.0048 (2)	−0.0041 (2)
O1	0.0258 (13)	0.0283 (15)	0.0280 (14)	−0.0073 (11)	0.0036 (11)	−0.0125 (11)
O2	0.0224 (13)	0.0206 (14)	0.0364 (15)	−0.0039 (10)	0.0047 (11)	−0.0022 (11)
O3	0.0470 (18)	0.0376 (17)	0.0378 (16)	−0.0199 (14)	0.0054 (14)	−0.0160 (13)
O4	0.0512 (19)	0.046 (2)	0.057 (2)	−0.0178 (15)	0.0228 (16)	−0.0343 (16)
O5	0.0226 (12)	0.0271 (14)	0.0222 (12)	−0.0056 (10)	−0.0001 (10)	−0.0089 (10)
O6	0.0211 (12)	0.0235 (14)	0.0258 (13)	−0.0055 (10)	0.0027 (10)	−0.0073 (10)
O7	0.0308 (14)	0.0296 (15)	0.0369 (15)	0.0100 (12)	−0.0139 (12)	−0.0169 (12)
O8	0.0222 (13)	0.0320 (15)	0.0250 (13)	−0.0017 (11)	−0.0061 (11)	−0.0095 (11)
O9	0.0349 (14)	0.0140 (12)	0.0215 (12)	−0.0062 (10)	−0.0067 (11)	−0.0016 (10)
O10	0.0352 (14)	0.0145 (12)	0.0207 (12)	−0.0031 (10)	−0.0032 (11)	−0.0002 (10)
O11	0.0367 (14)	0.0211 (13)	0.0190 (12)	0.0028 (11)	−0.0064 (11)	−0.0042 (10)
O12	0.0392 (15)	0.0133 (13)	0.0285 (14)	0.0033 (11)	−0.0062 (12)	−0.0056 (10)
O13	0.0287 (14)	0.0401 (17)	0.0280 (14)	−0.0078 (12)	−0.0103 (11)	0.0068 (12)
O14	0.0282 (14)	0.0287 (15)	0.0301 (14)	0.0032 (11)	−0.0076 (11)	−0.0084 (11)
O15	0.0264 (13)	0.0219 (13)	0.0304 (14)	−0.0060 (10)	0.0002 (11)	−0.0076 (11)
N1	0.0215 (15)	0.0211 (16)	0.0239 (15)	−0.0018 (12)	0.0000 (12)	−0.0070 (12)
N2	0.0182 (14)	0.0165 (14)	0.0220 (15)	0.0014 (11)	−0.0025 (12)	−0.0018 (11)
N3	0.0223 (14)	0.0156 (14)	0.0159 (14)	−0.0019 (11)	−0.0018 (11)	−0.0034 (11)
C1	0.0178 (16)	0.0178 (17)	0.0229 (17)	0.0008 (13)	−0.0037 (14)	−0.0049 (13)
C2	0.0240 (18)	0.0167 (17)	0.0295 (19)	−0.0045 (14)	−0.0036 (15)	−0.0030 (14)
C3	0.0252 (19)	0.024 (2)	0.029 (2)	−0.0025 (15)	−0.0016 (16)	−0.0078 (16)
C4	0.037 (2)	0.034 (2)	0.031 (2)	−0.0092 (18)	0.0076 (17)	−0.0137 (17)
C5	0.029 (2)	0.032 (2)	0.032 (2)	−0.0102 (17)	0.0068 (17)	−0.0101 (17)
C6	0.0179 (17)	0.0220 (18)	0.0242 (18)	0.0047 (14)	−0.0054 (14)	−0.0030 (14)
C7	0.035 (2)	0.022 (2)	0.029 (2)	−0.0021 (16)	−0.0053 (17)	−0.0090 (16)
C8	0.0175 (16)	0.0229 (19)	0.0233 (17)	−0.0030 (13)	−0.0005 (14)	−0.0022 (14)
C9	0.0218 (17)	0.0253 (19)	0.0202 (17)	0.0024 (14)	−0.0017 (14)	−0.0061 (14)
C10	0.0215 (17)	0.0199 (18)	0.0197 (17)	0.0032 (14)	−0.0074 (14)	−0.0040 (13)
C11	0.0157 (16)	0.0218 (18)	0.0229 (17)	−0.0012 (13)	−0.0040 (14)	−0.0040 (14)
C12	0.0167 (16)	0.0178 (17)	0.0187 (16)	0.0004 (13)	−0.0027 (13)	−0.0021 (13)
C13	0.0188 (16)	0.0173 (17)	0.0207 (17)	0.0024 (13)	−0.0050 (14)	−0.0037 (13)
C14	0.0253 (18)	0.0232 (18)	0.0147 (16)	0.0010 (14)	−0.0046 (14)	−0.0036 (13)
C15	0.0205 (16)	0.0133 (16)	0.0195 (16)	−0.0003 (13)	−0.0030 (13)	−0.0026 (13)
C16	0.0272 (18)	0.0164 (17)	0.0154 (16)	0.0005 (13)	−0.0056 (14)	−0.0010 (13)
C17	0.0216 (17)	0.0158 (17)	0.0208 (17)	0.0016 (13)	−0.0033 (14)	−0.0048 (13)
C18	0.040 (2)	0.0151 (17)	0.0200 (17)	−0.0041 (15)	−0.0046 (16)	−0.0028 (13)
C19	0.036 (2)	0.0182 (18)	0.0160 (16)	−0.0015 (15)	−0.0051 (15)	0.0006 (13)
C20	0.0169 (16)	0.0136 (16)	0.0220 (17)	−0.0005 (12)	0.0009 (13)	−0.0031 (13)
C21	0.0209 (17)	0.0193 (18)	0.0184 (16)	−0.0049 (13)	−0.0001 (14)	−0.0043 (13)

### Geometric parameters (Å, °)

**Table:**

Eu1—O6^i^	2.485 (2)	O15—H15B	0.8200
Eu1—O8	2.386 (3)	O15—H15A	0.8200
Eu1—O10^ii^	2.404 (2)	N1—C5	1.335 (5)
Eu1—O11^iii^	2.385 (2)	N1—C1	1.352 (4)
Eu1—O12^i^	2.328 (2)	N2—C8	1.341 (4)
Eu1—O13	2.454 (3)	N2—C12	1.346 (4)
Eu1—O14	2.442 (3)	N3—C15	1.348 (4)
Eu1—O15	2.510 (3)	N3—C19	1.327 (5)
Cu1—O1	1.932 (3)	C1—C6	1.517 (5)
Cu1—O5^iv^	1.944 (2)	C1—C2	1.374 (5)
Cu1—O7	2.359 (3)	C2—C3	1.392 (5)
Cu1—N1	1.975 (3)	C3—C7	1.528 (5)
Cu1—N2^iv^	1.975 (3)	C3—C4	1.392 (5)
Cu2—O2	2.409 (3)	C4—C5	1.382 (6)
Cu2—O9	1.968 (2)	C8—C9	1.377 (5)
Cu2—N3	1.969 (3)	C9—C10	1.389 (5)
Cu2—O2^ii^	2.409 (3)	C10—C11	1.393 (5)
Cu2—O9^ii^	1.968 (2)	C10—C14	1.505 (5)
Cu2—N3^ii^	1.969 (3)	C11—C12	1.376 (5)
O1—C6	1.283 (4)	C12—C13	1.516 (5)
O2—C6	1.227 (4)	C15—C20	1.520 (5)
O3—C7	1.242 (5)	C15—C16	1.375 (5)
O4—C7	1.242 (5)	C16—C17	1.389 (5)
O5—C13	1.272 (4)	C17—C21	1.508 (5)
O6—C13	1.242 (4)	C17—C18	1.388 (5)
O7—C14	1.251 (4)	C18—C19	1.381 (5)
O8—C14	1.251 (5)	C2—H2A	0.9300
O9—C20	1.261 (4)	C4—H4A	0.9300
O10—C20	1.241 (4)	C5—H5A	0.9300
O11—C21	1.260 (4)	C8—H8A	0.9300
O12—C21	1.242 (4)	C9—H9A	0.9300
O13—H13B	0.8200	C11—H11A	0.9300
O13—H13A	0.8200	C16—H16A	0.9300
O14—H14A	0.8200	C18—H18A	0.9300
O14—H14B	0.8200	C19—H19A	0.9300
			
O8—Eu1—O13	143.07 (9)	H15A—O15—H15B	99.00
O8—Eu1—O14	76.92 (8)	Eu1—O15—H15A	123.00
O8—Eu1—O15	75.96 (8)	Eu1—O15—H15B	122.00
O8—Eu1—O11^iii^	144.45 (9)	C1—N1—C5	118.9 (3)
O6^i^—Eu1—O8	68.55 (8)	Cu1—N1—C1	111.1 (2)
O8—Eu1—O12^i^	88.94 (8)	Cu1—N1—C5	130.0 (3)
O8—Eu1—O10^ii^	108.10 (8)	Cu1^iv^—N2—C8	128.5 (2)
O13—Eu1—O14	133.64 (9)	C8—N2—C12	119.2 (3)
O13—Eu1—O15	126.29 (9)	Cu1^iv^—N2—C12	112.3 (2)
O11^iii^—Eu1—O13	72.28 (9)	Cu2—N3—C19	128.7 (2)
O6^i^—Eu1—O13	75.67 (8)	Cu2—N3—C15	111.9 (2)
O12^i^—Eu1—O13	74.51 (10)	C15—N3—C19	119.4 (3)
O10^ii^—Eu1—O13	72.65 (10)	N1—C1—C6	114.2 (3)
O14—Eu1—O15	73.99 (8)	C2—C1—C6	123.6 (3)
O11^iii^—Eu1—O14	74.60 (9)	N1—C1—C2	122.2 (3)
O6^i^—Eu1—O14	124.22 (8)	C1—C2—C3	119.5 (3)
O12^i^—Eu1—O14	144.83 (9)	C2—C3—C4	117.7 (3)
O10^ii^—Eu1—O14	71.70 (9)	C2—C3—C7	120.2 (3)
O11^iii^—Eu1—O15	76.07 (8)	C4—C3—C7	122.0 (3)
O6^i^—Eu1—O15	132.71 (7)	C3—C4—C5	120.0 (3)
O12^i^—Eu1—O15	71.37 (9)	N1—C5—C4	121.7 (4)
O10^ii^—Eu1—O15	143.28 (8)	O1—C6—C1	115.5 (3)
O6^i^—Eu1—O11^iii^	146.55 (8)	O1—C6—O2	125.7 (3)
O11^iii^—Eu1—O12^i^	102.40 (8)	O2—C6—C1	118.8 (3)
O10^ii^—Eu1—O11^iii^	82.57 (8)	O3—C7—C3	116.6 (3)
O6^i^—Eu1—O12^i^	77.62 (8)	O3—C7—O4	126.3 (4)
O6^i^—Eu1—O10^ii^	79.02 (8)	O4—C7—C3	117.1 (3)
O10^ii^—Eu1—O12^i^	143.40 (10)	N2—C8—C9	121.5 (3)
O1—Cu1—O7	97.47 (10)	C8—C9—C10	119.7 (3)
O1—Cu1—N1	84.50 (11)	C9—C10—C11	118.4 (3)
O1—Cu1—O5^iv^	172.30 (10)	C9—C10—C14	120.4 (3)
O1—Cu1—N2^iv^	94.73 (11)	C11—C10—C14	121.1 (3)
O1—Cu1—O2^v^	92.14 (9)	C10—C11—C12	118.9 (3)
O7—Cu1—N1	93.91 (10)	C11—C12—C13	124.3 (3)
O5^iv^—Cu1—O7	90.08 (9)	N2—C12—C13	113.4 (3)
O7—Cu1—N2^iv^	93.32 (10)	N2—C12—C11	122.1 (3)
O2^v^—Cu1—O7	164.52 (8)	O5—C13—C12	115.7 (3)
O5^iv^—Cu1—N1	96.55 (10)	O6—C13—C12	118.4 (3)
N1—Cu1—N2^iv^	172.76 (12)	O5—C13—O6	125.9 (3)
O2^v^—Cu1—N1	99.09 (10)	O8—C14—C10	117.1 (3)
O5^iv^—Cu1—N2^iv^	83.28 (10)	O7—C14—O8	126.2 (3)
O2^v^—Cu1—O5^iv^	80.16 (8)	O7—C14—C10	116.7 (3)
O2^v^—Cu1—N2^iv^	73.73 (10)	N3—C15—C20	113.9 (3)
O2—Cu2—O9	88.97 (10)	C16—C15—C20	123.7 (3)
O2—Cu2—N3	88.99 (10)	N3—C15—C16	122.4 (3)
O2—Cu2—O2^ii^	180.00	C15—C16—C17	118.4 (3)
O2—Cu2—O9^ii^	91.03 (10)	C16—C17—C21	120.6 (3)
O2—Cu2—N3^ii^	91.01 (10)	C18—C17—C21	120.5 (3)
O9—Cu2—N3	83.49 (11)	C16—C17—C18	118.9 (3)
O2^ii^—Cu2—O9	91.03 (10)	C17—C18—C19	119.3 (3)
O9—Cu2—O9^ii^	180.00	N3—C19—C18	121.6 (3)
O9—Cu2—N3^ii^	96.51 (10)	O9—C20—C15	115.9 (3)
O2^ii^—Cu2—N3	91.01 (10)	O10—C20—C15	118.3 (3)
O9^ii^—Cu2—N3	96.51 (11)	O9—C20—O10	125.7 (3)
N3—Cu2—N3^ii^	180.00	O12—C21—C17	117.9 (3)
O2^ii^—Cu2—O9^ii^	88.97 (10)	O11—C21—O12	124.9 (3)
O2^ii^—Cu2—N3^ii^	88.99 (10)	O11—C21—C17	117.2 (3)
O9^ii^—Cu2—N3^ii^	83.49 (10)	C1—C2—H2A	120.00
Cu1—O1—C6	114.4 (2)	C3—C2—H2A	120.00
Cu2—O2—C6	120.1 (2)	C3—C4—H4A	120.00
Cu1^v^—O2—Cu2	138.98 (10)	C5—C4—H4A	120.00
Cu1^v^—O2—C6	94.1 (2)	N1—C5—H5A	119.00
Cu1^iv^—O5—C13	115.0 (2)	C4—C5—H5A	119.00
Eu1^i^—O6—C13	131.3 (2)	N2—C8—H8A	119.00
Cu1—O7—C14	130.9 (2)	C9—C8—H8A	119.00
Eu1—O8—C14	134.9 (2)	C8—C9—H9A	120.00
Cu2—O9—C20	113.9 (2)	C10—C9—H9A	120.00
Eu1^ii^—O10—C20	127.0 (2)	C10—C11—H11A	121.00
Eu1^vi^—O11—C21	125.3 (2)	C12—C11—H11A	121.00
Eu1^i^—O12—C21	173.8 (3)	C15—C16—H16A	121.00
H13A—O13—H13B	112.00	C17—C16—H16A	121.00
Eu1—O13—H13A	108.00	C17—C18—H18A	120.00
Eu1—O13—H13B	120.00	C19—C18—H18A	120.00
H14A—O14—H14B	104.00	N3—C19—H19A	119.00
Eu1—O14—H14A	105.00	C18—C19—H19A	119.00
Eu1—O14—H14B	114.00		
			
O13—Eu1—O8—C14	−178.0 (3)	Cu1—O7—C14—C10	−65.8 (4)
O14—Eu1—O8—C14	−27.5 (3)	Eu1—O8—C14—O7	31.0 (5)
O15—Eu1—O8—C14	49.0 (3)	Eu1—O8—C14—C10	−149.5 (2)
O11^iii^—Eu1—O8—C14	9.9 (4)	Cu2—O9—C20—O10	171.5 (3)
O6^i^—Eu1—O8—C14	−162.9 (3)	Cu2—O9—C20—C15	−10.4 (3)
O12^i^—Eu1—O8—C14	120.0 (3)	Eu1^ii^—O10—C20—O9	6.3 (5)
O10^ii^—Eu1—O8—C14	−93.0 (3)	Eu1^ii^—O10—C20—C15	−171.72 (19)
O8—Eu1—O11^iii^—C21^iii^	119.5 (3)	Eu1^vi^—O11—C21—O12	−20.8 (5)
O13—Eu1—O11^iii^—C21^iii^	−55.5 (3)	Eu1^vi^—O11—C21—C17	159.0 (2)
O14—Eu1—O11^iii^—C21^iii^	157.4 (3)	Cu1—N1—C1—C2	−179.0 (3)
O15—Eu1—O11^iii^—C21^iii^	80.5 (3)	Cu1—N1—C1—C6	3.7 (3)
O8—Eu1—O6^i^—C13^i^	152.9 (3)	C5—N1—C1—C2	1.9 (5)
O13—Eu1—O6^i^—C13^i^	−36.4 (3)	C5—N1—C1—C6	−175.5 (3)
O14—Eu1—O6^i^—C13^i^	97.0 (3)	Cu1—N1—C5—C4	−179.0 (3)
O15—Eu1—O6^i^—C13^i^	−163.0 (3)	C1—N1—C5—C4	0.0 (5)
O8—Eu1—O10^ii^—C20^ii^	−10.3 (3)	C12—N2—C8—C9	3.3 (5)
O13—Eu1—O10^ii^—C20^ii^	130.9 (3)	Cu1^iv^—N2—C8—C9	−175.7 (3)
O14—Eu1—O10^ii^—C20^ii^	−79.3 (3)	C8—N2—C12—C11	1.3 (5)
O15—Eu1—O10^ii^—C20^ii^	−100.9 (3)	C8—N2—C12—C13	−174.1 (3)
O7—Cu1—O1—C6	−96.6 (2)	Cu1^iv^—N2—C12—C11	−179.6 (3)
N1—Cu1—O1—C6	−3.3 (2)	Cu1^iv^—N2—C12—C13	5.0 (3)
N2^iv^—Cu1—O1—C6	169.5 (3)	Cu2—N3—C15—C16	−179.6 (3)
O2^v^—Cu1—O1—C6	95.6 (2)	Cu2—N3—C15—C20	1.9 (3)
O1—Cu1—O7—C14	−48.9 (3)	C19—N3—C15—C16	0.7 (5)
N1—Cu1—O7—C14	−133.9 (3)	C19—N3—C15—C20	−177.8 (3)
O5^iv^—Cu1—O7—C14	129.6 (3)	Cu2—N3—C19—C18	−177.5 (3)
N2^iv^—Cu1—O7—C14	46.3 (3)	C15—N3—C19—C18	2.2 (5)
O1—Cu1—N1—C1	−0.5 (2)	N1—C1—C2—C3	−2.1 (5)
O1—Cu1—N1—C5	178.5 (3)	C6—C1—C2—C3	175.1 (3)
O7—Cu1—N1—C1	96.6 (2)	N1—C1—C6—O1	−6.7 (4)
O7—Cu1—N1—C5	−84.4 (3)	N1—C1—C6—O2	170.7 (3)
O5^iv^—Cu1—N1—C1	−172.9 (2)	C2—C1—C6—O1	176.0 (3)
O5^iv^—Cu1—N1—C5	6.2 (3)	C2—C1—C6—O2	−6.7 (5)
O2^v^—Cu1—N1—C1	−91.8 (2)	C1—C2—C3—C4	0.4 (5)
O2^v^—Cu1—N1—C5	87.2 (3)	C1—C2—C3—C7	−174.7 (3)
O7—Cu1—O5^iv^—C13^iv^	−98.6 (2)	C2—C3—C4—C5	1.4 (6)
N1—Cu1—O5^iv^—C13^iv^	167.5 (2)	C7—C3—C4—C5	176.3 (4)
O1—Cu1—N2^iv^—C8^iv^	14.0 (3)	C2—C3—C7—O3	−6.4 (5)
O1—Cu1—N2^iv^—C12^iv^	−166.9 (2)	C2—C3—C7—O4	172.3 (4)
O7—Cu1—N2^iv^—C8^iv^	−83.8 (3)	C4—C3—C7—O3	178.8 (4)
O7—Cu1—N2^iv^—C12^iv^	95.3 (2)	C4—C3—C7—O4	−2.5 (6)
O1—Cu1—O2^v^—C6^v^	−12.3 (2)	C3—C4—C5—N1	−1.6 (6)
N1—Cu1—O2^v^—C6^v^	72.5 (2)	N2—C8—C9—C10	−4.7 (5)
O9—Cu2—O2—C6	158.3 (3)	C8—C9—C10—C11	1.4 (5)
O9—Cu2—O2—Cu1^v^	15.88 (14)	C8—C9—C10—C14	178.3 (3)
N3—Cu2—O2—C6	74.8 (3)	C9—C10—C11—C12	2.9 (5)
N3—Cu2—O2—Cu1^v^	−67.63 (15)	C14—C10—C11—C12	−173.9 (3)
O9^ii^—Cu2—O2—C6	−21.7 (3)	C9—C10—C14—O7	−37.3 (5)
N3^ii^—Cu2—O2—C6	−105.2 (3)	C9—C10—C14—O8	143.1 (3)
O2—Cu2—O9—C20	−80.0 (2)	C11—C10—C14—O7	139.4 (3)
N3—Cu2—O9—C20	9.1 (2)	C11—C10—C14—O8	−40.2 (5)
O2^ii^—Cu2—O9—C20	100.0 (2)	C10—C11—C12—N2	−4.4 (5)
N3^ii^—Cu2—O9—C20	−170.9 (2)	C10—C11—C12—C13	170.5 (3)
O2—Cu2—N3—C15	83.5 (2)	N2—C12—C13—O5	−0.9 (4)
O2—Cu2—N3—C19	−96.9 (3)	N2—C12—C13—O6	175.6 (3)
O9—Cu2—N3—C15	−5.6 (2)	C11—C12—C13—O5	−176.2 (3)
O9—Cu2—N3—C19	174.1 (3)	C11—C12—C13—O6	0.3 (5)
O2^ii^—Cu2—N3—C15	−96.5 (2)	N3—C15—C16—C17	−2.3 (5)
O2^ii^—Cu2—N3—C19	83.1 (3)	C20—C15—C16—C17	176.1 (3)
O9^ii^—Cu2—N3—C15	174.4 (2)	N3—C15—C20—O9	5.8 (4)
O9^ii^—Cu2—N3—C19	−6.0 (3)	N3—C15—C20—O10	−176.0 (3)
Cu1—O1—C6—O2	−171.0 (3)	C16—C15—C20—O9	−172.7 (3)
Cu1—O1—C6—C1	6.1 (4)	C16—C15—C20—O10	5.5 (5)
Cu2—O2—C6—O1	−76.7 (4)	C15—C16—C17—C18	1.0 (5)
Cu2—O2—C6—C1	106.3 (3)	C15—C16—C17—C21	−176.4 (3)
Cu1^v^—O2—C6—O1	79.6 (4)	C16—C17—C18—C19	1.7 (6)
Cu1^v^—O2—C6—C1	−97.4 (3)	C21—C17—C18—C19	179.1 (3)
Cu1^iv^—O5—C13—O6	179.9 (3)	C16—C17—C21—O11	−27.2 (5)
Cu1^iv^—O5—C13—C12	−3.9 (3)	C16—C17—C21—O12	152.6 (4)
Eu1^i^—O6—C13—O5	−4.7 (5)	C18—C17—C21—O11	155.5 (4)
Eu1^i^—O6—C13—C12	179.13 (19)	C18—C17—C21—O12	−24.7 (5)
Cu1—O7—C14—O8	113.8 (4)	C17—C18—C19—N3	−3.4 (6)

Symmetry codes: (i) −*x*+1, −*y*+1, −*z*; (ii) −*x*+1, −*y*+2, −*z*; (iii) *x*, *y*, *z*+1; (iv) −*x*, −*y*+1, −*z*; (v) −*x*, −*y*+2, −*z*; (vi) *x*, *y*, *z*−1.

### Hydrogen-bond geometry (Å, °)

**Table:**

*D*—H···*A*	*D*—H	H···*A*	*D*···*A*	*D*—H···*A*
O13—H13A···O5^i^	0.82	2.00	2.769 (3)	155
O13—H13B···O15^vii^	0.82	2.02	2.822 (4)	164
O14—H14A···O7	0.82	1.89	2.690 (4)	164
O14—H14B···O4^viii^	0.82	1.88	2.667 (4)	161
O15—H15A···O3^ix^	0.82	1.83	2.630 (4)	164
O15—H15B···O4^viii^	0.82	2.06	2.814 (4)	153
C18—H18A···O6	0.93	2.48	3.389 (4)	167

Symmetry codes: (i) −*x*+1, −*y*+1, −*z*; (vii) −*x*+1, −*y*+1, −*z*+1; (viii) −*x*, −*y*+2, −*z*+1; (ix) *x*, *y*−1, *z*.
